# Illuminating and Sniffing Out the Neuromodulatory Roles of Dopamine in the Retina and Olfactory Bulb

**DOI:** 10.3389/fncel.2020.00275

**Published:** 2020-08-31

**Authors:** Kirill S. Korshunov, Laura J. Blakemore, Paul Q. Trombley

**Affiliations:** ^1^Department of Biological Science, Florida State University, Tallahassee, FL, United States; ^2^Program in Neuroscience, Florida State University, Tallahassee, FL, United States

**Keywords:** dopamine, vision, olfaction, retina, olfactory bulb, circadian rhythms, biophysical properties, Parkinson’s disease

## Abstract

In the central nervous system, dopamine is well-known as the neuromodulator that is involved with regulating reward, addiction, motivation, and fine motor control. Yet, decades of findings are revealing another crucial function of dopamine: modulating sensory systems. Dopamine is endogenous to subsets of neurons in the retina and olfactory bulb (OB), where it sharpens sensory processing of visual and olfactory information. For example, dopamine modulation allows the neural circuity in the retina to transition from processing dim light to daylight and the neural circuity in the OB to regulate odor discrimination and detection. Dopamine accomplishes these tasks through numerous, complex mechanisms in both neural structures. In this review, we provide an overview of the established and emerging research on these mechanisms and describe similarities and differences in dopamine expression and modulation of synaptic transmission in the retinas and OBs of various vertebrate organisms. This includes discussion of dopamine neurons’ morphologies, potential identities, and biophysical properties along with their contributions to circadian rhythms and stimulus-driven synthesis, activation, and release of dopamine. As dysregulation of some of these mechanisms may occur in patients with Parkinson’s disease, these symptoms are also discussed. The exploration and comparison of these two separate dopamine populations shows just how remarkably similar the retina and OB are, even though they are functionally distinct. It also shows that the modulatory properties of dopamine neurons are just as important to vision and olfaction as they are to motor coordination and neuropsychiatric/neurodegenerative conditions, thus, we hope this review encourages further research to elucidate these mechanisms.

## Introduction

The central nervous system processes various stimuli, which allow it to respond to a constantly changing environment, while also contributing to the experiences that will allow an organism to adapt and survive. A crucial component for this to occur are neuromodulators, including dopamine. Dopamine is a famous neuromodulator that is most known for its role in rewards, addiction, motor control, and, to a lesser extent, its involvement with neurogenesis, daily rhythms, and the processing of sensory information. Dopamine is a catecholamine that is derived from the amino acid tyrosine, which is converted to L-DOPA via tyrosine hydroxylase (TH, the rate-limiting enzyme in dopamine production); L-DOPA is then converted to dopamine via aromatic L-amino acid decarboxylase. Dopamine can also be converted to norepinephrine (via dopamine-β-hydroxylase) followed by epinephrine (via phenylephanolamine N-methyltransferase), the other two major catecholamines of the central and peripheral nervous systems. Over the last few decades, there has been a tremendous growth in understanding of the role of dopamine in various systems, including its regulation of two of the most crucial senses for vertebrate and invertebrate organisms: vision and olfaction.

Vision arises from responding to the electromagnetic stimuli that first hit the retina, and olfaction occurs when deciphering a volatile milieu of chemical odorants that are detected by olfactory sensory neurons (OSNs) and then processed by the olfactory bulb (OB). There are 11 catecholamine-expressing nuclei in the brain (Felten and Sladek, 1983; Hökfelt et al., 1984), and the retina and OB possess their own endogenous dopamine neuron populations. In the retina, the modulatory dopamine interneurons assist the photoreceptors, and nearly all other retinal neurons, in transitioning from processing scotopic light (during the nighttime) to processing photopic light (during the daytime). In the OB, the number of endogenous dopamine neurons may outnumber all other dopaminergic populations in the vertebrate brain (Cave and Baker, 2009). Like the retina, the dopaminergic OB neurons are also modulatory interneurons, which help gate certain odor stimuli and increase odor discrimination (the ability to tell one odor apart from another). The main goal of this review is to explore the dopamine neuron populations in the retina and OB and their neuromodulatory mechanisms. To provide the most comprehensive view of these neurons, we also explore their identities and morphologies, their daily rhythms, activity-dependent expression, biophysical properties, and the potential disruption of their neuromodulatory mechanisms in neurodegenerative diseases such as Parkinson’s disease (PD). These findings also provide insight into the many remarkable similarities and differences between the retina and OB, thus, vision and olfaction. While we focus on the neuromodulatory roles of dopamine specifically in vertebrate organisms (fish, amphibians, turtles, reptiles, mice, rats, rabbits, cats, and people), there is also a rich literature on the activity of dopamine in various insect species, particularly in their olfactory systems (Perk and Mercer, 2006; Dacks et al., 2012; Boto et al., 2014; Lizbinski and Dacks, 2018; Sayin et al., 2018).

**FIGURE 1:**
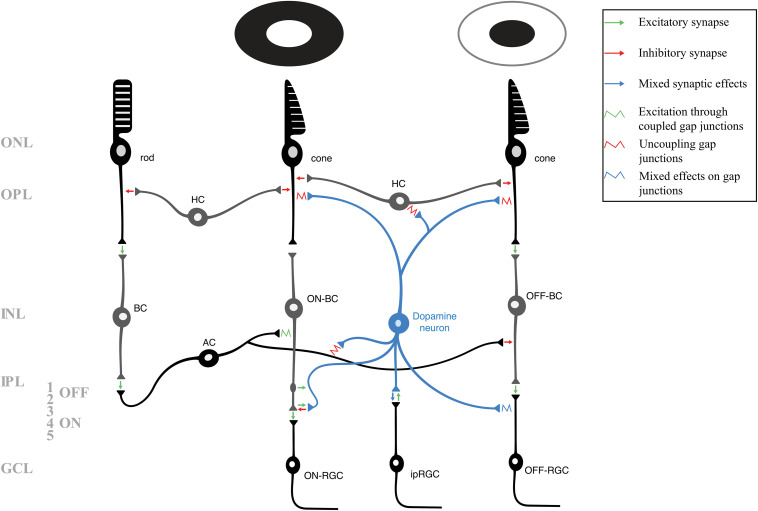
A schematic of the layers and neuronal circuitry of the retina, including the pathways of rods, cones that respond to light (middle photoreceptor), and cones that respond to dark (right photoreceptor). For clarity and simplicity, many neurons and synapses have been excluded. The various modulatory mechanisms of retinal dopamine neurons (blue) affect nearly every retinal neuron to allow the retina to adapt to photopic conditions. Green arrows indicate excitatory (glutamatergic) synapses, red arrows indicate inhibitory (GABAergic/glycinergic) synapses, and blue arrows indicate mixed synaptic effects. A potential excitatory *en passant* synapse (between ON-bipolar cell axon and dopamine neuron) is shown in strata 1 of the inner plexiform layer. Retinal gap junctions, which are also targets of various dopaminergic and other modulatory mechanisms, are represented by squiggles. The green squiggle indicates depolarization via heterotypic coupling, red squiggles indicate dopaminergic uncoupling of gap junctions, and blue squiggle indicates mixed effects of dopamine on the coupling or uncoupling of gap junctions. AC, amacrine cell; BC, bipolar cell (including those depolrized – ON – and inhibited – OFF – by light); GCL, ganglion cell layer; HC, horizontal cell; INL, inner nuclear layer; IPL, inner plexiform layer (including the OFF – 1 and 2 – and ON – 3, 4, and 5 – strata); ipRGC, intrinsically photosensitive retinal ganglion cell; ONL, outer nuclear layer; OPL, outer plexiform layer; RGC, retinal ganglion cell (including the ON and OFF-RGCs).

**FIGURE 2:**
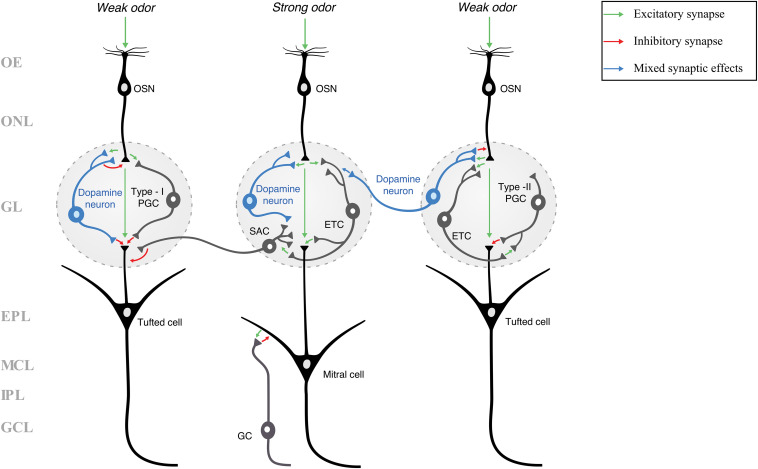
A schematic of the layers and neuronal circuitry of the olfactory bulb (many neurons and synapses have been excluded for clarity and simplicity). Because the neuronal identity of dopamine neurons is not agreed upon, they are classified in this figure simply as “dopamine neurons” and not as a specific type of juxtaglomerular cell (e.g., PGC or SAC). Green arrows indicate excitatory (glutamatergic) synapses, red arrows indicate inhibitory (GABAergic and/or dopaminergic) synapses, and blue arrows indicate mixed synaptic effects (inhibition through GABA, followed by an increased likelihood for excitation by dopamine). Three pathways are shown, each either receiving a weak or a strong odor stimulus. A weak odor or artificial stimulus is hypothesized to activate dopamine neurons (blue neurons), while a strong odor stimulus is hypothesized to inactivate dopamine neurons (Ennis et al., 2001; Korshunov et al., 2020). In our previous work (Korshunov et al., 2020), we showed that rat olfactory bulb dopamine neurons are more responsive (produced more action potentials) to weak rather than strong current stimuli, potentially increasing the release of dopamine and/or GABA, resulting in presynaptic inhibition (McGann, 2013). Dopamine activity within fish olfactory bulbs was shown to reduce the transmission of weak stimuli while strong stimuli were processed more than weak stimuli (Bundschuh et al., 2012). Thus, the dopamine neuron in the center glomerulus does not provide presynaptic inhibition or other modulation in response to a strong odor stimulus, while the dopamine neurons in the left and right glomeruli respond to weak stimuli with inhibitory or mixed synaptic effects. In showing potential dopaminergic synaptic effects, this schematic illustrates one of the potential mechanisms of lateral inhibition and odor discrimination. EPL, external plexiform layer; ETC, external tufted cell; GC, granule cell; GCL, granule cell layer; GL, glomerular layer; IPL, internal plexiform layer; MCL, mitral cell layer; OE, olfactory epithelium; ONL, olfactory nerve layer; OSN, olfactory sensory neuron; PGC, periglomerular cell; SAC, short-axon cell.

To understand the context of dopamine’s activity, it is necessary to introduce their laminar organization and neuronal populations of the retina (Figure 1) and the OB (Figure 2). These structures have also been reviewed in detail elsewhere (Witkovsky, 2004; Ennis et al., 2007, 2015; Nagayama et al., 2014; Kosaka and Kosaka, 2016; Roy and Field, 2019). Light first activates the rod and cone photoreceptors in the outer retina, specifically in the outer nuclear layer (ONL_R_), from which rods and cones transduce and transmit light information to the excitatory bipolar cells (BCs) and the inhibitory horizontal cells (HCs) in the outer plexiform layer (OPL). (Please note that some of the abbreviations between retinal and OB layers are the same. To avoid confusion, the identical abbreviations related to the retina will have the “R” subscript, and an “OB” subscript will be included for OB layers. e.g., ONL_R_ and ONL_OB_.) In photopic conditions, rods and certain cones activate the ON-BCs, while the OFF-BCs will activate in scotopic conditions by another subset of cones and indirectly by rods. The BCs send this light information along the inner nuclear layer (INL) to the dendrites of the retinal ganglion cells (RGCs) and a subset of RGCs called the intrinsically photosensitive retinal ganglion cells (ipRGCs). Synapses between BCs and RGCs form in the inner plexiform layer (IPL) – in the OFF portion (strata 1 and 2) for OFF-driven RGCs and in the ON portion (strata 3–5) for the ON-driven RGCs. Lastly, the transmission from BCs can be modulated by the inhibitory receptive field of the amacrine cells (ACs), either in the OFF or ON portion of the IPL. The RGCs and ipRGCs somas are in the deepest retinal layer, the ganglion cell layer (GCL_R_). From there, RGCs send light information to a few dozen nuclei (Dhande and Huberman, 2014), including those in the thalamus, hypothalamus, midbrain, and visual cortex as the optic nerve.

While visual processing in the retina ends with the optic nerve, odor processing begins with the olfactory nerve. Odor transduction begins in the olfactory epithelium, where OSNs are activated by a specific chemical odorant, allowing them to send an electrical impulse to the OB via the olfactory nerve. The OSNs axons pass through the first layer of the OB, the olfactory nerve layer (ONL_OB_), and terminate in the glomeruli of the glomerular layer (GL) where they form synapses with various OB neurons. Glomeruli are dense, neuropil-rich structures that are surrounded and innervated by a population of inhibitory interneurons – periglomerular cells (PGCs) and short-axon cells (SACs) – and excitatory interneurons – external tufted cells (ETCs). Collectively, these neurons are known as juxtaglomerular cells (JGCs), and they are the first neurons to have the opportunity to modulate the excitatory/glutamatergic odor signals received from the OSNs. These signals are picked up by the apical dendrites of the main output neurons of the OB – the mitral and tufted cells (M/TCs; these neurons are often abbreviated together due to their similar morphologies and proximity to one another, often making them difficult to differentiate in many studies). The processes and cell bodies of M/TCs span a number of OB layers, ranging from apical dendrites extending to the GL, to tufted cells’ somas localized to the external plexiform layer (EPL), and mitral cells’ somas localized deeper in the mitral cell layer (MCL). Further modulation of odor information occurs at dendrodendritic synapses between lateral dendrites of M/TCs and dendrites of inhibitory granule cells (GCs) in the EPL. Axons of the M/TCs extend to the granule cell layer (GCL_OB_) and then send signals from the OB, via the lateral olfactory tract, to various regions of the olfactory cortex and the limbic system.

Dopaminergic neurons in the retina and OB are mainly localized to the INL and the GL, respectively (Witkovsky, 2004; Ennis et al., 2007, 2015; Nagayama et al., 2014; Roy and Field, 2019). Their strategic localization allows them to affect sensory processing through various neuromodulatory mechanisms by activating specific dopamine receptors. Dopamine receptors are G-protein coupled receptors that fall into two categories: D_1_- and D_2_-like receptors. The D_1_ receptor family, which includes D_1_ and D_5_ receptors (D_1_R and D_5_R, respectively), acts to increase protein kinase A (PKA) activity by activating adenylyl cyclase via Gα_S_, which then increases cAMP production, and thus, the phosphorylation activity of PKA (Missale et al., 1998). The D_2_ receptor family, which includes D_2_, D_3_, and D_4_ receptors (D_2_R, D_3_R, and D_4_R, respectively), works in the opposite manner, where they inhibit the adenylyl cyclase/cAMP/PKA pathway via the Gα_i_ protein (Missale et al., 1998). The D_1_, D_2_, and D_4_Rs are found within the retinas of various species (Nguyen-Legros et al., 1996, 1997; Veruki and Wässle, 1996; Jackson et al., 2009; Li et al., 2013). The D_3_R gene (*Dr3d*) is not found in the retina, and the D_5_R gene (*Dr5d*), while apparently present, does not have a known protein expression or function in the retina (Jackson et al., 2009). The OBs of various species express D_1_Rs and D_2_Rs (Levey et al., 1993; Coronas et al., 1997; Duchamp-Viret et al., 1997; Koster et al., 1999; Gutièrrez-Mecinas et al., 2005; Rodrigues et al., 2014), but the presence of D_3_-D_5_Rs is not known.

We begin this review by first describing the morphology and neuronal identity of retinal and OB dopamine neurons.

## Morphology and Neuronal Identity

### Retinal Dopamine Neurons

The endogenous dopamine neurons of the retina, found within the INL, project their processes through the ON and OFF strata of the IPL and are most commonly referred to as ACs (Witkovsky, 2004; Roy and Field, 2019). There are many subpopulations of the retinal ACs (Roy and Field, 2019), with most recent estimates being around 140 cell types in the mouse (Yan et al., 2020), and the putative dopaminergic neurons were calculated to only comprise 0.08% of all of the mammalian ACs (Jeon et al., 1998). However, dopamine neurons are also sometimes classified as interplexiform cells, because their processes can extend to the outer retina (Versaux-Botteri et al., 1984; Wulle and Schnitzer, 1989; Umino and Dowling, 1991; Harsanyi and Mangel, 1992; Witkovsky et al., 2000). A similar identity crisis is associated with the dopaminergic neurons in the OB (discussed in section “Olfactory Bulb Dopamine Neurons”). To avoid any nomenclatural confusion, we will simply refer to these as retinal and OB dopamine neurons.

In the retina, the catecholamine/TH-expressing neurons can be divided into two groups: Type-1 and Type-2 cells. The Type-1 cells have a larger soma than Type-2s, they are localized within the INL, and have a high expression of TH (Versaux-Botteri et al., 1984; Mariani and Hokoc, 1988; Tauchi et al., 1990; Wang et al., 1990; Zhang et al., 2004). Soma sizes of Type-1 cells appear consistent across mammalian species: roughly 12.5 to 15.5 μm in cats (Wang et al., 1990) and 13.5 μm in mice (Zhang et al., 2004). Type-1 cells have thick and thin processes that originate from the soma and extend toward stratum 1 of the IPL, and they also have fine processes that extend toward the OPL (Zhang et al., 2004). Type-2 cells are distinctly different from the Type-1 cells. Type-2 cells have smaller somas, are often more widespread in their localization (they are found in the INL, IPL, and GCL_R_), they are more numerous, and have a dimmer staining for TH than Type-1 cells (Versaux-Botteri et al., 1984; Mariani and Hokoc, 1988; Tauchi et al., 1990; Wang et al., 1990; Zhang et al., 2004). Type-2 cell processes arborize in the middle of the IPL (Mariani and Hokoc, 1988). During development, the number of Type-1 cells (∼4,000 in the cat’s retina) remains the same before and after eyes open, with their dendritic appendages growing until postnatal day 13 (when cats’ eyes begin to open) (Wang et al., 1990). Conversely, the smaller Type-2 cells become dimmer and lose TH expression throughout development, and their density also appears to drop from ∼40,000 neurons at postnatal day 1 to ∼7,400 neurons when eyes open at postnatal day 13, although this is likely due to decreased TH expression rendering them undetectable (Wang et al., 1990). Thus, these two types of catecholaminergic retinal neurons may serve different roles in modulation of retinal neurons, as well as playing some role during development. Presently, only the Type-1 cell is considered to be dopaminergic.

Some recent and classic studies comment on the consistently low expression of TH in Type-2 cells, or describe simply not being able to label them with the TH antibody in transgenic animals (Versaux-Botteri et al., 1986; Zhang et al., 2004; Contini et al., 2010). Zhang et al. (2004) noted that a reason for this could be that the TH promoter was more sensitive than the antibody used in detecting TH. It was speculated that the Type-2 cells are actually epinephrine neurons (Versaux-Botteri et al., 1986), which may be present in the mammalian retina (Hadjiconstantinou et al., 1983). Also, Type-2 cells did not appear to have an axon, while Types-1 cells did (Zhang et al., 2004). Consistent with the idea of there being at least two types of retinal TH neurons, a recent study found that a specific subset of ACs (C25) express TH at higher levels than other types of ACs (Yan et al., 2020). Additionally, these C25 TH neurons (presumably the Type-1 dopamine neurons) expressed two isoforms of the precursor enzyme to γ-amino butyric acid (GABA): high levels of glutamic acid decarboxylase-67 (GAD-67) and moderate levels of GAD-65 (Yan et al., 2020). These data provide the general distinction that Type-1 cells are dopaminergic, while the nature of the Type-2 catecholamine cells remains to be elucidated. While this may give the impression that Type-1 retinal dopamine neurons are a homogenous population, they were later differentiated by their different stimulus-dependent and biophysical activities (discussed in section “Light-Driven Activation and Synthesis of Retinal Dopamine” and section “The Spiking Profile of Retinal Dopamine Neurons,” respectively).

### Olfactory Bulb Dopamine Neurons

Localized almost entirely to the GL, there are ∼100,000–150,000 dopamine neurons in the OB of the adult rat and ∼89,000 dopamine neurons in the OB of the adult mouse, which correspond to 10-16% of all JGCs being dopaminergic (McLean and Shipley, 1988; Panzanelli et al., 2007; Parrish-Aungst et al., 2007). Dopamine neurons are also found in the OBs of humans (Smith et al., 1993; Alizadeh et al., 2015). These dopamine neurons, subtypes of PGCs and GCs, are continuously generated in the subventricular zone (SVZ) and migrate to the OB throughout adulthood (McLean and Shipley, 1988; Gross, 2000; Lledo et al., 2006; Merkle et al., 2007; Whitman and Greer, 2007; Pignatelli et al., 2009; Galliano et al., 2018). The implication of these adult-born neurons is that they could be used as therapeutic treatment for PD (Baker et al., 2001; Alizadeh et al., 2019).

While the retinal dopamine neurons are mostly classified as ACs (and, to a lesser extent, as interplexiform cells or interplexiform ACs), the identity of OB dopamine neurons is less settled. Classically, these neurons were identified as ETCs (Halász et al., 1981; Davis and Macrides, 1983). However, this classification is no longer used because ETCs are glutamatergic and excitatory (Hayar et al., 2004), while OB dopamine neurons (like retinal dopamine neurons: Wulle and Wagner, 1990; Contini and Raviola, 2003; Hirasawa et al., 2009; Yan et al., 2020) express GAD-67 and GABA and are thus inhibitory (Kosaka et al., 1985, 1987, 1995; Gall et al., 1987; Baker, 1990; Wilson and Wood, 1992; Kosaka and Kosaka, 2007; Maher and Westbrook, 2008; Kiyokage et al., 2010; Borisovska et al., 2013; Liu et al., 2013). Currently, OB dopamine neurons are typically classified as either PGCs or SACs.

The PGC is the most ubiquitous and widely used classification of OB DA neurons (Kosaka and Kosaka, 2011, 2016). PGCs are the most numerous and smallest of the JGCs – with somas ranging from 5 to 10 μm (Ennis et al., 2007; Nagayama et al., 2014). Like the retinal catecholamine neurons, these PGCs consist of at least two types: Type-1 and Type-2 PGCs. The putative PGCs that receive synaptic input from OSNs and expresses TH are classified as the Type-1 PGCs (Kosaka et al., 1997, 1998; Kosaka and Kosaka, 2004, 2007). The PGCs that do not receive input from OSNs and do not express TH, but do express Ca^2+^-binding proteins such as calretinin, calbindin, and parvalbumin, are classified as Type-2 PGCs (Kosaka et al., 1997, 1998; Kosaka and Kosaka, 2004, 2005). Our own immunolabeling results (unpublished data) confirm that rat OB dopamine neurons do not express calretinin. Thus, if a subset or all of the OB dopamine neurons are the PGCs, then they may be the Type-1, but not the Type 2 PGCs.

In the previous decade, it was common to identify OB dopamine neurons as SACs, largely due the fact these neurons have long, interglomerular (contacting multiple neighboring and distant glomeruli) axonic processes (Kiyokage et al., 2010; Liu et al., 2013, 2016). In fact, dopamine neurons are the most common source of interglomerular projections in the OB (Kosaka and Kosaka, 2008), with some projections spanning up to 1 mm (Aungst et al., 2003; Kiyokage et al., 2010). These neurons’ dendritic processes can also project to and ramify in 7 to 39 glomeruli (Kiyokage et al., 2010). However, there is argument that OB dopamine neurons cannot all be SACs because most of these neurons do not have an axon (Chand et al., 2015; Galliano et al., 2018), and their dendritic branching is more attributed to the PGC and not the SAC morphology (Kosaka and Kosaka, 2009, 2011, 2016; Kosaka et al., 2019).

While the identity of the dopaminergic OB JGCs remains to be determined, there is near universal agreement that these neurons fall into at least two subpopulations. These subpopulations are typically differentiated by a bimodal distribution of soma size and diameter (Pignatelli et al., 2005; Kosaka and Kosaka, 2007, 2008, 2009, 2011), by the presence or absence of an axon (Chand et al., 2015; Galliano et al., 2018), and by the difference in their biophysical properties, such as action potential spiking and the Na^+^ current (I_Na_) (Korshunov et al., 2020). However, the OB dopamine populations may be more heterogenous than simply two subtypes. A recent study by Kosaka et al. (2019) presented evidence that there may be four or more different types of dopaminergic JGCs. These include the “Large PGCs,” which are axonic and have dendrites with few spines that would tuft in one or several glomeruli, which may be the subpopulation that was previously classified as SACs (Kosaka et al., 2019). The neurons classically thought of as being PGCs were redefined as the “Small PGCs,” which had small soma diameters, typically lacked an axon, and had 1–4 spiny dendritic processes that could tuft in as many glomeruli (Kosaka et al., 2019). The “Transglomerular” neurons displayed a dendrite that spanned up to 6 glomeruli, and the “Incrusting” cells are the smaller dopamine neurons that had a mostly non-spiny dendrite spanning the periphery of its glomerulus (Kosaka et al., 2019). Furthermore, some of the examined dopamine neurons did not fit into any of the above categories, leaving them unclassified (Kosaka et al., 2019).

## Circadian Rhythms

Daily rhythms that oscillate in a 24-h period are called circadian rhythms (Latin for “about a day”). Circadian rhythms regulate nearly all biological functions, including gene transcription, metabolism and body temperature, hunger, neuronal activities, and many others, by synchronizing them to a specific time of the day. Zeitgebers, including light and temperature, are the external cues that entrain (set phase to) these rhythms. Once thought of as the master circadian pacemaker, the suprachiasmatic nucleus (SCN) of the hypothalamus sets the rhythmicity of the brain and body by receiving light signals from the melanopsin-expressing ipRGCs via the retinohypothalamic tract (Mohawk et al., 2012). While the SCN is capable of maintaining rhythmicity through a transcriptional inhibitory feedback loop of the canonical “clock” genes/proteins (discussed further, but see Mohawk et al., 2012 for more details), it is now thought that peripheral clocks in cellular populations are capable of operating independently as long as they receive entrainment information from zeitgebers (Husse et al., 2015). Normally functioning cellular clocks allow biological processes to correspond with the time of day (e.g., higher metabolism in the daytime/afternoon, lower body temperature during the night). Areas such as the retina and OB are unique because they have an “inner/autonomous clock” that can maintain rhythmicity in the absence of zeitgebers and a functioning SCN.

In this section, we explore the established and the proposed roles of retinal and OB dopamine neurons, respectively, in contributing to their circadian rhythms and inner clocks. It should also be noted that when studies describe the circadian rhythms of the animal or a tissue, that these rhythms are often recorded under constant darkness, without light acting to entrain the rhythmicity. “Diurnal” rhythms, on the other hand, are often recorded in some presence of light, typically in 12-h light/dark cycles.

### Circadian Rhythms in the Retina and Dopamine’s Involvement

Given that the retina is crucial for sending signals to and entraining the SCN, it is not surprising that it is necessary for it to maintain an autonomous inner clock. Early evidence of this clock was presented in cultured amphibian and mammalian retinas, where serotonin N-acetyltransferase (NAT, one of the key enzymes in the production of melatonin) and melatonin exhibited circadian rhythmicity (Besharse and Iuvone, 1983; Iuvone, 1986; Tosini and Menaker, 1996). In amphibian retinal cultures, circadian melatonin production is dependent on the presence of rods and cones (Cahill and Besharse, 1993), and dopamine, acting through the D_2_R, suppressed melatonin production in these cultures (Cahill et al., 1991). This inhibitory action was confirmed to be present in mammalian retina, where the D_2_/D_4_R, present on photoreceptors, inhibited melatonin synthesis (Nguyen-Legros et al., 1996; Tosini and Dirden, 2000). The mechanism likely involves D_2_R activation decreasing cAMP activity, which, in turn, decreases NAT activity and melatonin production (Iuvone, 1986). Thus, the inhibitory feedback loop between retinal melatonin and dopamine is a crucial mechanism for the maintenance of circadian rhythms in the retina (Tosini et al., 2008).

**FIGURE 3:**
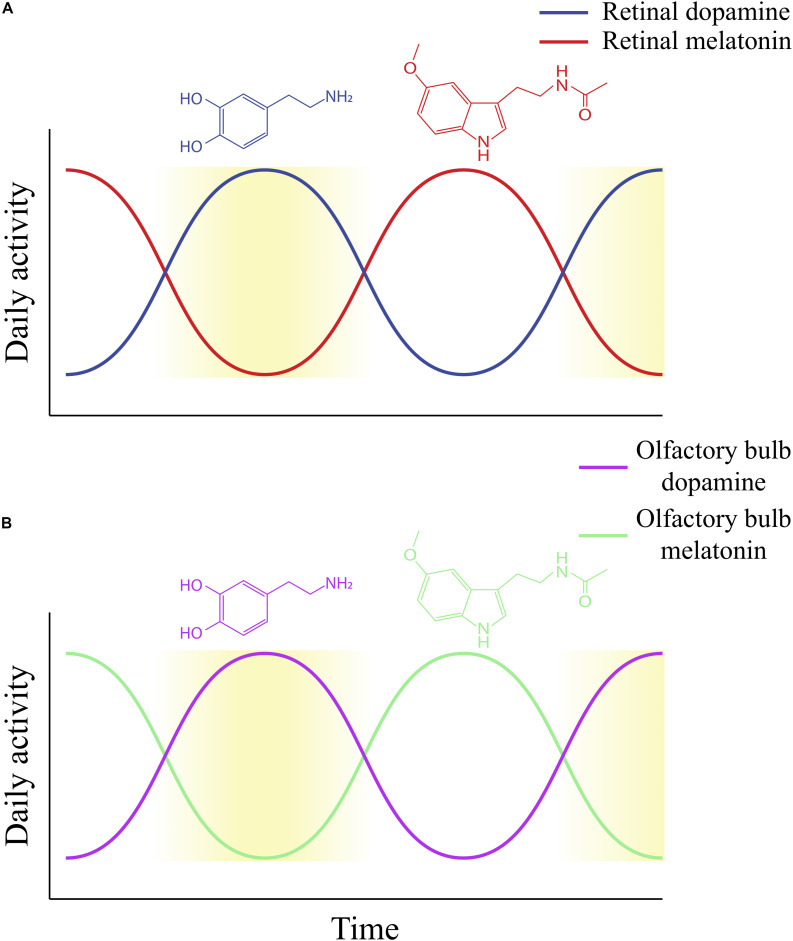
The daily rhythms of dopamine and melatonin activity, as established within the retina **(A)** and proposed within the olfactory bulb **(B)**. Increasing yellow gradient indicates an increase in the level of light to which the retina is exposed **(A)** or is an indicator of the time of day for the olfactory bulb **(B)**. **(A)** Retinal dopamine synthesis and activity is highest during daytime, while retinal melatonin activity is highest during nighttime. This figure does not make the distinction between light-driven and circadian dopamine synthesis and release. While both would produce similar rhythms (dopamine is highest during the daytime or subjective day), dopamine release in constant darkness (circadian) would be much lower than light-stimulated dopamine release. **(B)** The proposed daily rhythm of dopamine in the olfactory bulb based on our prior study on dopamine content and release over 24 h in a12-h light/dark cycle. We found that olfactory bulb dopamine demonstrates diurnal activity, with highest activity occurring when lights are on and lowest levels when lights are off (Corthell et al., 2013). The proposed daily melatonin rhythm in the olfactory bulb is based on our prior determination of levels of mRNA for melatonin synthesizing enzymes in rats exposed to 12-h light/dark cycles. We found that HIOMT mRNA, for example, fluctuates in a diurnal fashion, with the lowest expression during lights-on and highest expression during lights-off (Corthell et al., 2014). Thus, this figure illustrates potential diurnal rhythms of olfactory bulb dopamine and melatonin, which peak in the daytime and nighttime, respectively. However, it has not been determined whether olfactory bulb dopamine and melatonin display circadian rhythms.

In vertebrate retinas, synthesis and activity of dopamine increase during daytime as melatonin simultaneously falls. The presence of retinal melatonin rhythms is necessary for dopamine to maintain circadian rhythms in mammalian (Doyle et al., 2002a), fish (Ribelayga et al., 2004), and reptilian retinas (Bartell et al., 2007). In contrast to results of previous studies, the rhythmicity of dopamine was found to not be dependent upon the presence of rod and cone photoreceptors, as mutant rats with degenerated photoreceptors still maintained rhythmicity of dopamine activity up to 2 weeks in constant darkness (Doyle et al., 2002b). However, this is likely due to the finding that retinal melatonin can adapt to conditions where photoreceptor functionality is lost (Tosini et al., 2008). In addition to being expressed in photoreceptors, NAT mRNA is also expressed in the inner retina (INL and GCL_R_) (Liu et al., 2004) at low levels, but becomes upregulated in dystrophic retinas where photoreceptors are degenerated (Sakamoto et al., 2004). These and other mechanisms (possibly including melatonin supplied by the pineal gland) could be contingencies in order to maintain the autonomous clock of dystrophic retinas. Thus, the circadian release of retinal melatonin is needed to maintain the circadian rhythms of dopamine (Tosini et al., 2008). The relative circadian rhythms of melatonin and dopamine are summarized in Figure 3A. When light acts as a stimulus, it increases retinal dopamine levels above the circadian levels that would be seen in constant darkness (the response of dopamine neurons to light is discussed in section “Light-Driven Activation and Synthesis of Retinal Dopamine”).

In mammals, HCs, dopaminergic and catecholaminergic ACs, and ipRGCs all possess mRNAs of the six main clock genes that regulate circadian rhythms through the inhibitory transcription/translation feedback loop: *Clock*, *Bmal1*, *Cry1*, *Cry2*, *Per1*, and *Per2* (Ruan et al., 2006). In addition, the protein CRY1 is found to be expressed in every retinal layer and CRY2 is expressed in photoreceptors (Wong et al., 2018). One study shows that these genes are most expressed within the dopaminergic neurons and are completely absent from the photoreceptors (Ruan et al., 2006). However, another study shows that these genes are expressed by and show circadian transcriptional activity in photoreceptors (Dkhissi-Benyahya et al., 2013). This discrepancy may be due to the finding that cone photoreceptors express clock proteins with circadian rhythmicity, while rod photoreceptors do not (Liu et al., 2012). Thus, the autonomous clock of the retina is present in the INL, GCL_R_, and likely the OPL (Witkovsky et al., 2003; Ruan et al., 2006; Dkhissi-Benyahya et al., 2013). The presence of the clock in the INL was further supported because this explanted layer still shows a rhythmic expression of the PER2 protein (Ruan et al., 2008).

All retinal dopaminergic receptors impact some aspect of circadian rhythms. Activation of D_1_Rs phase-advances PER2 expression during the subjective day (when an animal is in constant darkness, its rhythms still oscillate, with certain peaks occurring during its subjective “day” and “night”) and phase-delays the expression during the subjective night (Ruan et al., 2008). Activation of D_2_Rs, in turn, increases the activity of the BMAL1:CLOCK complex, which is then necessary to transcribe the *Per1* gene in the retina (Yujnovsky et al., 2006). D_4_R activation modulates the rhythmic expression of another clock gene, *Npas2*, in RGCs, which is thought to increase daytime contrast sensitivity (ability to detect variations in light intensities) (Hwang et al., 2013), though it’s not clear if RGCs themselves express the D_4_R. In the ipRGCs, D_2_R activation is necessary for the regulation of the mRNA of the photopigment melanopsin (Sakamoto et al., 2005).

### Circadian Rhythms in the Olfactory Bulb and Dopamine’s Potential Involvement

Analogous to the retina, the OB also possess its own autonomous inner clock. Perhaps the earliest evidence of this was observed when cultured neurons from rat OBs exhibited strong rhythmic expression of the *Per1* gene, without input from the SCN, which peaked during the nighttime (Abe et al., 2002). These results were strengthened by the persistence of *Per1* rhythmicity in the presence of constant light and an abolished SCN (Granados-Fuentes et al., 2004a). The rhythmic activity of *Per1* was shown to be intrinsic to mitral and (possibly) tufted cells, suggesting that the main output neurons of the OB possess the internal clock (Granados-Fuentes et al., 2004b). Circadian expression of c-Fos, a marker of neuronal activity, was also induced in the OB layers and the piriform cortex (PC, part of the olfactory cortex) in response to odor, even with an abolished SCN (Granados-Fuentes et al., 2006). Those data also showed that the OB, not the SCN, was responsible for maintaining the c-Fos rhythmicity in the PC, and olfactory responsivity would be highest at night (Granados-Fuentes et al., 2006, 2011). Olfactory discrimination itself was shown to be rhythmic, with highest sensitivity being at early night, and was dependent on the presence of *Bmal1* and *Per1/2* (Granados-Fuentes et al., 2006). *Per1* expression in the OB also increases from day to night, corresponding to increasing discrimination (Abraham et al., 2005).

Unlike the retina, the known mechanisms that contribute to the autonomous clock in the OB are relatively scant. It can be speculated, however, that retaining the oscillation of odor functions such as discrimination, threshold perception, and odor-evoked behavior (e.g., foraging, predator avoidance) are important for increasing the chance of survival of vertebrates that depend on odor as much as humans depend on vision. This would especially make sense for nocturnal rodents, whose olfactory sensitivity would need to be highest during dusk or nighttime. Dopamine is known to impact olfactory discrimination (Tillerson et al., 2006; Escanilla et al., 2009). However, it is not known what impact dopamine may have on the circadian rhythms of the OB. Our group had previously found that OB dopamine demonstrates diurnal activity, with highest activity occurring when lights are on and lowest levels when lights are off (Corthell et al., 2013). This may suggest that either these dopamine neurons have an autonomous rhythm or that their rhythms are regulated by other neurons, possibly the M/TCs (Granados-Fuentes et al., 2004b). A neuropeptide called vasoactive intestinal polypeptide (VIP) may contribute to dopamine rhythms. VIP, acting through its receptor VPAC_2_, is crucial for the rhythmic expression of PER2 and odor detection (Miller et al., 2014). Because VIP itself is expressed in the GL and the EPL, and its receptor is expressed by M/TCs and ETCs (Miller et al., 2014; and our own unpublished findings), it may be possible that VIP directly or indirectly influences the rhythmicity of dopamine neurons and, perhaps, all other JGCs in the glomeruli.

Additionally, our group showed that melatonin receptors and another one of its biosynthesis enzymes, hydroxyindole-O-methyltransferase (HIOMT), is found in the OB (Corthell et al., 2014). The receptor and enzyme mRNAs fluctuate in a diurnal fashion, with HIOMT showing the lowest expression during lights-on and highest expression during lights-off (Corthell et al., 2014). We also showed that melatonin receptor activation impacts the biophysical activity of JGCs, including decreasing their outward K^+^ current (I_K_) (Corthell et al., 2014), affecting those neurons’ excitability. While it is not known if melatonin is produced in the OB endogenously, these results imply that melatonin and its receptors may contribute the autonomous clock of the OB. Furthermore, given that dopamine and melatonin provide inhibitory feedback to each other in the retina, they may display a similar activity in the OB (this rhythm is proposed in Figure 3B). These are some of the avenues we plan to further study to better identify the mechanisms of OB circadian rhythms.

## Stimulus-Dependent Activation

### Light-Driven Activation and Synthesis of Retinal Dopamine

It is universally accepted that retinal dopamine levels and activity increase with light (Figure 3A). This increase in dopamine allows the retina to transition from scotopic, rod-driven processing to photopic, cone-driven processing (Witkovsky, 2004; Roy and Field, 2019). Dopamine synthesis and activity spikes in response to light onset (Nir et al., 2000a). Neuronal activity in response to white light (Godley and Wurtman, 1988) also increases with the light intensity (Brainard and Morgan, 1987). An increase in light-driven phosphorylation of TH corresponds to the increase in dopamine synthesis (Iuvone, 1984). Light activity or activation of D_1_R increases phosphorylation of TH and also increases c-Fos labeling in the TH/dopaminergic neurons (Koistinaho and Sagar, 1995). The specific TH sites that are phosphorylated by light (as well as glutamate- and acetylcholine- mediated activation) are serine residues 19, 31, and 40 (Witkovsky et al., 2000). Increasing spiking activity also increases the phosphorylation of TH at these three sites, and vice versa (Witkovsky et al., 2004).

In bright light, activated ON-BCs excite retinal dopamine neurons. The “stratification rule” of the IPL dictates that ON-BCs would excite dopamine neurons in the ON sublamina of the IPL, which was confirmed to occur in stratum 3 of the IPL (Contini et al., 2010). However, evidence also exists that ON-BCs excite retinal dopamine neurons in the OFF sublamina (stratum 1) of the IPL, breaking this rule (Dumitrescu et al., 2009; Hoshi et al., 2009). It is possible that retinal dopamine neurons receive two excitatory inputs from the ON-BCs: via an *en passant* synapse in the OFF sublamina and through a classical synapse of the ON sublamina. Interestingly, the excitatory input from ON-BCs may not be needed to drive this phosphorylation/dopamine synthesis (Witkovsky et al., 2000). This result may suggest that other sources, including the input from ipRGCs (Zhang et al., 2008; Prigge et al., 2016), are also driving this activity.

However, despite a previous report (Doyle et al., 2002b), even further evidence shows that photoreceptors are necessary for dopamine synthesis and activity. Mice that carry a mutation that causes them to lose the outer segment of their photoreceptor (retinal dystrophy) with age show significantly lower levels of dopamine synthesis compared to wildtypes (Nir and Iuvone, 1994; Nir et al., 2000b). This effect is more pronounced in homozygotes (all photoreceptors degenerated) than heterozygotes (half photoreceptors present) (Nir et al., 2000b). Yet, a relatively high steady state level of dopamine, just not dopamine synthesis, is still retained in these retinas (Nir and Iuvone, 1994). It can also be considered that melatonin – which is higher during the nighttime (Figure 3A) and has an antagonistic interplay with dopamine (Iuvone, 1986), but for which dopamine is also dependent upon to have functional circadian rhythms (Doyle et al., 2002a; Ribelayga et al., 2004; Bartell et al., 2007) – is present even in the absence of functional photoreceptors, but its rhythms are abolished (Tosini and Menaker, 1998). Activation driven by ipRGCs can be considered as an alternative pathway to rod and cone photoreceptors, but this is further complicated by new findings that argue that rod-activated BCs are the sole source of excitation for the retinal dopamine neurons (Munteanu et al., 2018; Pérez-Fernández et al., 2019). Thus, further research is needed to fully understand all the light-driven activation pathways of retinal dopamine neurons.

Lastly, light influences the biophysical activity of dopaminergic neurons in different ways, providing insight that this neuronal population is not as homogenous as expected. Some retinal dopamine neurons produce a transient (brief, burst at the onset of the stimulus) response to light, others produce a sustained (similar spike frequency throughout the duration of the stimulus) response, while others produce no response to light at all (maintain their spiking frequency before and after the light stimulus) (Zhang et al., 2007, 2008). These findings and their implications are further discussed in section “The Spiking Profile of Retinal Dopamine Neurons.”

### Stimulus-Evoked Synthesis of Dopamine in the Olfactory Bulb

Deep to the GCL_OB_ is the subependymal layer (Ennis et al., 2007, 2015). This layer receives a large number of adult-born GCs that migrate to the deeper layers of the OB (GCL_OB_, IPL_OB_, and MCL), dopamine neurons, and other JGCs that mostly migrate to the GL (Luskin, 1993; Ennis et al., 2007, 2015). The migration of these progenitor neurons is very much an active process, as some 10,000 new interneurons enter the mouse OB every day (Nagayama et al., 2014). Adult-born progenitor neurons migrate from the SVZ to the OB via the rostral migratory stream (RMS). Once migrated to the OB, if the immature neurons express the transcription factors Pax6 and Olig2 (Hack et al., 2005) and are able to receive excitatory synaptic input, they can differentiate into the dopaminergic phenotype.

There is a wealth of findings that show that when an animal’s nasal cavity is obstructed from receiving odors (naris occlusion), which leads to a decrease in OSN stimulation of the ipsilateral OB hemisphere, the OB dopamine neurons decrease their expression of TH (Baker et al., 1983; Baker, 1990; Wilson and Wood, 1992; Wilson and Sullivan, 1995), D_2_Rs increase their density in the ONL and GL (Guthrie et al., 1991), and the M/TCs become more responsive to odor stimuli (Guthrie et al., 1990). The decrease in TH expression was originally thought to not be indicative of dopaminergic cell death, because L-amino acid decarboxylase (the enzyme that converts L-DOPA to dopamine) was still expressed during naris occlusion (Baker et al., 1984), suggesting only a decrease in dopamine synthesis. However, more recent studies show that dopamine density decreases following prolonged (1–4 weeks) naris occlusion (Sawada et al., 2011; Grier et al., 2016). This loss is likely driven by microglia, which became activated during naris occlusion and appeared to engulf dopaminergic synapses (Grier et al., 2016). Subsequent naris reopening was shown to increase the number of newborn OB dopamine neurons (Sawada et al., 2011). Thus, the survival of OB dopamine neurons depends on OSN input (Sawada et al., 2011). TH expression also appears to be governed by OSN input, likely through the activation of the NMDA glutamate receptors (Puche and Shipley, 1999) and the L-type Ca^2+^ channel (Cigola et al., 1998).

It was originally thought that OB dopamine neurons only express TH once they reach the GL (McLean and Shipley, 1988), where they form excitatory synapses with OSNs’ terminals and the apical dendrites of M/TCs. Both OSNs (Berkowicz et al., 1994; Ennis et al., 1996) and M/TCs (Trombley and Westbrook, 1990) release glutamate at these synapses. However, subsequent findings indicated that TH expression is also present in layers deep to the GL, including the EPL, MCL, and GCL (Baker et al., 2001; Pignatelli et al., 2009; Kosaka et al., 2019; Korshunov et al., 2020). There is no conclusive finding to explain why TH expression can occur deep to the GL, but Pignatelli et al. (2009) had found that even ∼75% of the deep (in the EPL) TH green fluorescent protein (GFP) neurons are capable of responding to OSN stimulation. Other possible reasons may include that these neurons also receive glutamatergic input from the lateral dendrites of M/TCs. Or, they may be in proximity to glutamate spillover, also from the M/TCs. In all cases, there can be several pathways in which glutamate can activate the NMDA receptors and eventually the L-type Ca^2+^ channels, leading to increased TH expression. All possibilities seem likely, because even the deep TH-GFP neurons (whether in the EPL or the MCL) show an excitatory response to glutamate application (Pignatelli et al., 2009).

This population of OB dopamine neurons deep to the GL was classified as “immature” by some investigators (Pignatelli et al., 2009). Pignatelli et al. (2009) found that deep dopamine neurons in the EPL and MCL possess a larger intracellular Cl^–^ concentration than those within the GL. A high intracellular Cl^–^ concentration is a hallmark of immature neurons (Ben-Ari et al., 2007), making this classification enticing. However, it is unclear whether their high Cl^–^ concentration is a function of immaturity or is a marker of a particular type of OB dopamine neuron. Further research is needed to answer the question as to whether these deep dopamine neurons are immature.

## Biophysical Properties

### The Spiking Profile of Retinal Dopamine Neurons

The biophysical properties of dopamine (and other) neurons include action potential spiking, activation of ionic currents, and signal facilitation. These properties show how dopamine neurons behave spontaneously and how they respond to stimuli (whether natural or artificial). These intrinsic biophysical activities can provide insight into how retinal and OB neurons influence their neuronal circuitry. In the absence of synaptic input, cultured retinal dopamine neurons showed spontaneous spiking activity within the θ-frequency (less than ∼10 Hz or spikes per second) (Gustincich et al., 1997; Feigenspan et al., 1998). This spiking frequency increases with additional depolarizing current and with kainate (glutamate receptor agonist) application, and it is conversely blocked by GABA and D_2_R activation (Gustincich et al., 1997; Puopolo et al., 2001). This action potential spiking in cultured retinal dopamine neurons appears to be required for the vesicular release of dopamine (Puopolo et al., 2001). Thus, dopamine activity and release may increase above background/spontaneous activity in the daytime through increased ON-BC (and from other sources) excitation in the daytime, and decrease through increased inhibitory input from ACs (which would likely be stimulated by the OFF-BCs) during the nighttime (Gustincich et al., 1997; Puopolo et al., 2001; Zhang et al., 2007). Indeed, Zhang et al. (2007) confirmed that GABAergic and glycinergic antagonists increased spontaneous spiking and more instances of bursting from dopamine neurons in the dark. These effects of GABA are likely most prevalent during darkness in the nighttime (as opposed to darkness stimuli in the daytime), because melatonin may act through GABA_A_R to inhibit dopamine neurons (Boatright et al., 1994). Therefore, these neurons display spontaneous spiking that is synaptically regulated, with increased excitatory drive in the daytime and increased inhibition in the night.

As mentioned in section “Light-Driven Activation and Synthesis of Retinal Dopamine,” retinal dopamine neurons display heterogenous spiking activities in response to light. Recordings from mouse dopaminergic neurons in retinal slices show that, surprisingly, not all neurons respond to light (Zhang et al., 2007). Only 60% of the recorded neurons responded (increase in spike frequency) to light, while the rest did not respond (but they still showed spontaneous activity) (Zhang et al., 2007). The dopamine neurons that did respond to light were further differentiated by their specific activities: a majority produced a transient response (rapid bursting of action potentials at the onset of the light stimulus, followed by a decrease in spiking frequency), and the minority displayed a sustained response (action potential frequency increased to light onset, and maintained this frequency throughout the duration of the stimulus) (Zhang et al., 2007). These “transient” and “sustained” groups of dopamine neurons were most responsive to wavelengths of green, then blue, and least responsive to red light (Zhang et al., 2007). These spiking properties are modulated by synaptic excitation and inhibition (Zhang et al., 2007), so these groups of dopamine neurons likely receive different synaptic input in order to generate this difference in spiking properties. While the transient group is likely driven by ON-BCs, the sustained group was determined to be driven by ipRGCs (Zhang et al., 2007, 2008).

Because the melanopsin-expressing ipRGCs are considered to be the “third” photoreceptor of the retina (Berson et al., 2002; Hattar et al., 2002), and because they provide glutamatergic input to retinal dopamine neurons (Zhang et al., 2012; Prigge et al., 2016; Liu et al., 2017), it is likely that they are responsible for driving the sustained light response in dopamine neurons that is independent of the transient light response driven by cone ON-BCs. By blocking out the ON-BC input via L-AP4, an agonist for the metabotropic glutamate receptor mGluR6, which blocks transmission from photoreceptors onto ON-BCs, the sustained group of dopamine neurons was effectively isolated (Zhang et al., 2008). These neurons responded most to blue wavelength (∼478 nm), which is the wavelength to which the melanopsin in ipRGCs is most responsive (Lucas et al., 2001; Hattar et al., 2003; Zhang et al., 2008). Conversely, transient neurons responded more to the 500 nm wavelength (Zhang et al., 2008), which is the wavelength to which the opsin photopigments of rods and middle (green) wavelength cone photoreceptors are most responsive (Jacobs et al., 1991). This sustained response was preserved in retinas with degenerated rod and cone photoreceptors (Zhang et al., 2008). c-Fos labeling was also found in dopaminergic neurons and ipRGCs following light stimulation (while simultaneously blocking the cone and rod photoreceptor pathways with L-AP4) (Zhang et al., 2008). Thus, the group of dopamine neurons that respond to light in a sustained manner depend on light signaling from ipRGCs (Zhang et al., 2008). Subsequent studies have also established a glutamatergic, presynaptic input from ipRGCs to dopaminergic neurons (Zhang et al., 2012; Prigge et al., 2016; Liu et al., 2017). Lastly, without functional melanopsin in ipRGCs, the light-evoked increase in TH mRNA, which is normally present in wildtype retinas, is absent in these modified retinas (Dkhissi-Benyahya et al., 2013).

### The Spiking Profile of Olfactory Bulb Dopamine Neurons

Many key findings that have provided important insight into the biophysical properties of OB dopamine neurons have come with the advent of transgenic mouse models with neurons that express GFP linked to TH, making these fluorescent dopamine neurons an easy target for electrophysiology recordings. The most distinctive feature of these OB dopamine neurons was their spontaneous activity (Pignatelli et al., 2005, 2009, 2013; Puopolo et al., 2005). Like in the retina, OB dopamine neurons’ spiking was within the θ-frequency (∼8 Hz) in OB slices (Pignatelli et al., 2005). Also like in the retina (Puopolo et al., 2001), action potentials likely increase vesicular dopamine release (Borisovska et al., 2013). Interestingly, some of the aforementioned deep dopamine neurons also displayed this spontaneous spiking (specifically those recorded from the EPL), while other deeper neurons (recorded in the MCL) did not (Pignatelli et al., 2009). When these dopamine neurons were dissociated, their spiking increased to slightly above the θ-frequency (∼13 Hz) than what was seen in slice (Pignatelli et al., 2005), indicating that these neurons likely receive some inhibition in slice. This spontaneous activity was found to be driven by the TTX-sensitive persistent I_Na_, the T-type Ca^2+^ current (I_Ca_), and the hyperpolarization-activated cation h-current (I_H_) (Pignatelli et al., 2005, 2013; Puopolo et al., 2005).

We recently published findings on the biophysical properties of rat OB dopamine neurons, and described how these properties may distinguish different groups of dopamine neurons (as was discussed in section “Olfactory Bulb Dopamine Neurons”) (Korshunov et al., 2020). Similar to previous work in mice, we used a transgenic rat model (Iacovitti et al., 2014) with dopamine neurons that expressed a fluorescent protein to easily target cells. Recording from rat OB TH-GFP neurons also provided the opportunity to identify potential species differences between rats and mice. For example, none of the neurons from which we recorded displayed spontaneous spiking, only showing synaptically driven potentiation; this indicates a potential functional difference in the background activity between rat and mouse OB dopamine neurons, suggesting a possible functional and species difference (Pignatelli et al., 2005; Puopolo et al., 2005; Korshunov et al., 2020). We also found that, when these neurons were grouped based on their neuronal areas (in which we classified them as either “Large” or “Small” dopamine neurons), the Large dopamine neurons produced more action potentials in response to weak current stimuli, while the Small neurons only produced a more phasic/single spiking response to these stimuli (Korshunov et al., 2020). When stimulated with stronger current stimuli, the Large and Small neurons both produced the same phasic/single spiking response (Korshunov et al., 2020). This difference in responses was likely due to inactivation properties of the voltage-gated Na^+^ (Na_V_) channels. In Small neurons, Na_V_ channels were significantly more inactivated than those of Large neurons, especially at slightly depolarized membrane potentials (−70 and −60 mV), which likely drives the increased responsiveness of Large but not Small neurons to weak stimuli (Korshunov et al., 2020). Furthermore, Small neurons displayed a significantly stronger I_H_ than Large neurons, which could further inactivate their Na_V_ channels by increasing their resting membrane potentials (Korshunov et al., 2020).

These putative “Large” dopamine neurons are likely ones that possess an axon, while the “Small” neurons do not (Chand et al., 2015; Galliano et al., 2018). These axonic, Large dopamine neurons are overall more excitable than their anaxonic counterparts: they spike at more hyperpolarized membrane potentials, have faster spike onset, and spike at a higher frequency than the Small neurons in OB cultures (Chand et al., 2015) and slices (Galliano et al., 2018). Thus, the presence of an axon can be indicative of the excitability profile of the Large OB dopamine neurons (Chand et al., 2015; Galliano et al., 2018). These excitability profiles can contribute to the functional hypothesis of OB dopamine serving as a high-pass filter. We discuss this further in the “Neuromodulation” section “Dopamine’s Neuromodulation of the Olfactory Bulb.”

## Neuromodulation

### Dopamine’s Neuromodulation of the Retina

The daytime increase in dopamine allows the retina to adapt to light by increasing visual acuity and contrast sensitivity through various modulatory mechanisms (Witkovsky, 2004; Roy and Field, 2019). Dopamine modulates virtually every neuron in the retina, ultimately modulating the activity and receptive fields of RGCs. While many of these effects occur with dopamine acting directly on the synapses of retinal neurons, many of these changes also occur by modulating the gap junction proteins in between retinal neurons (Figure 1). Gap junctions are composed of homomeric or heteromeric connexin proteins that couple cells together, allowing for quick, electrical impulses to travel from one cell to another without a conventional chemical synapse. Different modulatory mechanisms can phosphorylate or dephosphorylate gap junctions, causing them to either close (uncouple) or open (couple) their connexin protein channels. When two cells of the same type are connected by a gap junction (e.g., rod-rod), they form a homotypic connection, while a heterotypic connection forms when gap junctions connect two different cells (e.g., BC-AC). The various neuromodulatory mechanisms of dopamine, beginning with photoreceptors, are summarized below. These effects (and the species in which they were analyzed) are also summarized in Table 1.

**TABLE 1:** Neuromodulatory effects of dopamine receptors on retinal neurons.

Cell type	D_1_	D_2_/D_2_-like receptor	D_4_
Photoreceptors	–	• Decreases rod-rod, rod-cone, cone-cone coupling (mice) • Decreases the I_*H*_ in rods (amphibians and humans) • Decreases melatonin production (amphibians) • Inhibits the Na^+^/K^+^ ATPase activity (rats)	• Inhibits melatonin synthesis (rat) • Decreases cAMP cascade (mice) • Decreases rod-cone coupling (mice and fish)
HCs	• Uncouples gap junctions (fish and primates)	• Indirectly couples gap junctions through the autoreceptor inhibition of dopamine release (fish)	–
BCs	• Decreases Na_*V*_ activity of ON-BCs (mice)	–	–
	• Helps decrease the spatial inhibition of rod- and OFF-BCs by inhibiting ACs (mice)		
ACs	• Uncouples gap junctions (rabbits)	–	–
RGCs	• Couples homotypic (RGC-RGC) and heterotypic (RGC-AC) gap junctions (mice and rabbits) • Decreases I_*Na*_ amplitude (fish and rats), and decreases action potential spiking (rats)	• Uncouples gap junctions (rabbits) • Decreases I_*K*_ and increases I_*Na*_ (rats)	• Modulates NPAS2 and adenylyl cyclase 1 activity, leading to increased contrast sensitivity (Not clear if D_4_Rs expressed by RGCs) (mice)
ipRGCs	• Attenuates melanopsin-based photocurrent (rats)	• Regulates the expression of melanopsin mRNA (rats)	–

*The “D_2_-like” column represents effects that could either be from D_2_ or D_4_Rs. No reported effects of D_3_ or D_5_Rs were found. The “–” indicates that there is not a known effect of the D_1_, D_2_, or D_4_Rs on the particular cell type. All references for listed effects are described in section “Dopamine’s Neuromodulation of the Retina.” ACs, amacrine cells; BCs, bipolar cells; HCs, horizontal cells; ipRGCs, intrinsically photosensitive retinal ganglion cells; RGCs, retinal ganglion cells.*

Photoreceptors must regulate their gap junctions’ coupling in order to adjust to the environmental lighting. During the night, photoreceptors are coupled, which helps increase the signal-to-noise ratio in low lighting, while they are uncoupled during the day (Tessier-Lavigne and Attwell, 1988; DeVries et al., 2002; Jin et al., 2015). The main connexin gap junctions that are present in mammalian and non-mammalian vertebrate photoreceptors are connexin 36 (Cx36) (O’Brien et al., 2012; Li et al., 2014). Dopamine is one of the main modulators of photoreceptor coupling. In the daytime, when its levels are high, dopamine decreases coupling between rods-cones, cones-cones, and rods-rods by activating the D_2_-like/D_4_Rs present on these photoreceptors (Ribelayga et al., 2008; Li et al., 2013; Jin et al., 2015). Activation of D_2_-like/D_4_Rs decreases the adenylyl cyclase/cAMP activity (Nir et al., 2002; Jackson et al., 2009, 2011; Hwang et al., 2013), which eventually dephosphorylates the Cx36 and uncouples it between the photoreceptors (Li et al., 2013). Interestingly, this mechanism also occurs in a circadian fashion, during the subjective day but in the absence of light (Ribelayga et al., 2008). This occurs because, during the subjective day, the antagonistic actions of retinal melatonin are low, leading to an increase in dopamine release (Ribelayga et al., 2008). The D_2_-like receptors have a higher affinity for dopamine than D_1_Rs, and because dopamine is released in a circadian fashion without additional release in response to light stimuli, these relatively low levels of dopamine are enough to uncouple photoreceptors via D_2_-like receptors in photoreceptors (Ribelayga and Mangel, 2003; Ribelayga et al., 2008). By uncoupling photoreceptors, dopamine also helps increase the contrast sensitivity (the ability to contrast between variations of light intensities) (Jackson et al., 2012). Besides gap junctions, dopamine directly influences the excitability of rods by inhibiting their Na^+^/K^+^ ATPase activity via the D_2_-like/D_4_Rs (Shulman and Fox, 1996). Dopamine also inhibits the I_H_ in rods through a D_2_-like/D_4_R activation in amphibian (Akopian and Witkovsky, 1996) and human retinas (Kawai et al., 2011). While it can be assumed that a decrease in I_H_ would decrease the excitability of rods (Kawai et al., 2011), further details are needed.

In the INL, the inhibitory HCs are coupled during the night and uncoupled during the day by dopamine (Roy and Field, 2019). Specifically, HCs are uncoupled by the activation of D_1_Rs (Harsanyi and Mangel, 1992; Wang et al., 1997; Ribelayga and Mangel, 2003; Zhang et al., 2011). Unlike the circadian-driven activation of the D_2_-like/D_4_Rs that uncouples photoreceptors, light is needed to release higher levels of dopamine to uncouple HCs by activating the low-affinity D_1_Rs (Ribelayga and Mangel, 2003). Conversely, the circadian-driven dopamine release at night (though its basal levels are still lower than daytime) may still partially uncouple the HCs, as there is evidence that shows that the D_1_R antagonist (SCH-23390) was capable of increasing the receptive field of HCs in the absence of light (Zhang et al., 2011). As HCs provide an antagonistic receptive field to the surrounding photoreceptors and the excitatory BCs (Roy and Field, 2019), daytime dopamine activity would decrease this inhibitory receptive field by uncoupling HCs, which could increase visual acuity (how accurately an object can be visually discerned) (Jackson et al., 2012). On the other end, activation of the D_2_Rs indirectly increases HC coupling, because D_2_Rs are expressed as autoreceptors on the dopaminergic neurons (at least in fish retinas) (Harsanyi and Mangel, 1992; Wang et al., 1997). Activation of the D_2_ autoreceptors would decrease dopamine release, thus, maintaining/increasing coupling of HCs (Harsanyi and Mangel, 1992; Wang et al., 1997).

Retinal dopamine affects the signal input onto RGCs by directly and indirectly modulating the activity of various BCs. D_1_R activation decreases the excitability of ON-BCs by decreasing their Na_V_ conductance when these neurons are activated by a light stimulus during the night (Smith et al., 2015). Dopamine is effective at decreasing ON-BC excitation when these neurons are presented with the light stimulus during night but not during day, likely in order to decrease ON-BC saturation in response to light (Smith et al., 2015). D_1_R activation can also decrease the light-evoked inhibition of rod BCs by presynaptically inhibiting the inhibitory activity of ACs (Flood et al., 2018). Likewise, cone OFF-BCs become inhibited by GABA and glycine released from ACs in lighted conditions (Mazade and Eggers, 2016). However, long light stimuli eventually decrease the spatial inhibition of ACs onto OFF-BCs, allowing them to adapt to the lighting condition (Mazade and Eggers, 2016). Part of this light-adapted decrease in inhibition is due to the activation of D_1_Rs, which presynaptically decrease the release of glycine from ACs (Mazade et al., 2019). Beyond D_1_R activity, other mechanisms likely contribute to the light adaptation of BCs.

Dopamine affects the coupling between the AII class of ACs, which may increase spatial acuity in bright lighting (Demb and Singer, 2012; Roy and Field, 2019). In moderate lighting (e.g., moonlight), the AII ACs receive excitatory input from rod BCs and transfer this signal via homotypic coupling between other AIIs toward the cone-driven ON-BCs via heterotypic coupling, thus, integrating the rod pathway into the cone pathway (Demb and Singer, 2012). AII ACs express D_1_Rs (Nguyen-Legros et al., 1997; but see also: Veruki and Wässle, 1996). D_1_R activation decreases homotypic coupling between the AII ACs (Hampson et al., 1992; Kothmann et al., 2009). At photopic light, this reduced coupling may increase acuity by decreasing the receptive fields of inner retinal neurons (BCs, ACs, and RGCs) that receive coupled excitatory or synaptic inhibitory (glycinergic) input from the AII ACs (Demb and Singer, 2012). Dopaminergic retinal neurons further affect the AII ACs by releasing GABA onto the AC somas, likely under the same lighting conditions at which dopamine is released (Contini and Raviola, 2003). This GABAergic inhibition from dopamine neurons also affects the ON-BCs (which provide the excitatory input onto the phasic group of dopamine neurons – Zhang et al., 2007) in the ON (stratum 3) portion of the IPL (Contini et al., 2010). These presynaptic inhibitory mechanisms of AIIs and ON-BCs are proposed to decrease noise from the rods during photic illumination (Contini et al., 2010).

Lastly, dopamine has numerous effects on the RGCs and the ipRGCs, which affects their coupling and biophysical activity. Light and D_1_R activation increases the homotypic coupling between the α group of OFF-RGCs and the heterotypic coupling between α OFF-RGCs and ACs (Hu et al., 2010). Conversely, D_2_R activation uncoupled the α OFF-RGCs (Mills et al., 2007). The implications of these effects are not clear (Roy and Field, 2019), but increased heterotypic and homotypic coupling does increase synchronous activity of the α OFF-RGCs (Hu et al., 2010), which would impact the visual signal transmission being sent via the optic nerve. Much is also understood about how dopamine affects the biophysical properties of RGCs and ipRGCs. In RGCs, D_1_R activation decreases the I_Na_ amplitude, modulates these neurons’ I_H_, and overall decreases their spiking (Hayashida and Ishida, 2004; Chen and Yang, 2007; Hayashida et al., 2009). Conversely, D_2_R activation decreases the I_K_ and increases the I_Na_ in these neurons, likely increasing overall excitability (Yin et al., 2020). Likewise, dopamine decreases the photocurrent of the ipRGCs via D_1_R, thus reducing the light-driven spiking, but also decreases the light-independent background spiking (Van Hook et al., 2012).

These results paint a complex, yet a profound picture of how dopamine can mediate the transition from retinal daytime to nighttime activity.

### Dopamine’s Neuromodulation of the Olfactory Bulb

Dopamine acts on the first synaptic contact in the glomeruli of the OB, where it can modify the transmission of the odor signal (Figure 2). The neuromodulatory effects of dopamine are mediated through the D_1_ and D_2_Rs, which are summarized (along with the specific species) in Table 2. To the best of our knowledge, no expression nor activity of D_3_, D_4_, or D_5_Rs have been found in the OB. The activity of OB D_2_Rs has been implicated in the modulation of odor discrimination (the ability to differentiate between two different odors) (Tillerson et al., 2006; Taylor et al., 2009), odor detection (Doty and Risser, 1989), and neonatal odor preference training (Coopersmith et al., 1991). During naris occlusion, which causes TH and dopamine to decrease, the M/TCs become more responsive to odor (Guthrie et al., 1990; Wilson and Sullivan, 1995) and OSN stimulation (Wilson and Wood, 1992), suggesting a decrease in odor discrimination. This effect was mimicked with application of the D_2_R antagonist spiperone in non-occluded OBs (Wilson and Sullivan, 1995). In another study, genetically modified mice lacking the D_2_R investigated novel odors significantly less than their wildtype counterparts, also indicating a decrease in discrimination (Tillerson et al., 2006). Additionally, the D_2_R agonist quinpirole decreased odor detection and discrimination in a dose-dependent manner (Doty and Risser, 1989; Escanilla et al., 2009). These last results may imply that activating D_2_Rs at high/saturating levels by agonists would decrease signal transmission from OSNs onto JGCs and/or M/TCs, thus decreasing odor discrimination due to a high detection threshold. Alternatively, inactivation of D_2_Rs by antagonists may decrease lateral glomerular inhibition, causing for more noise to be transmitted, and thus, for odor discrimination to also decrease due to a low signal-to-noise ratio. Therefore, to maintain proper levels of odor discrimination, certain glomeruli would need to be inactivated by dopamine while others would need to transmit the signal.

**TABLE 2:** Neuromodulatory effects of dopamine receptors on olfactory bulb neurons and astrocytes.

Cell type	D_1_R	D_2_R
OSNs	–	• Inhibits glutamate release (turtles, mice, and rats)
PGCs	–	• May act as an autoreceptor
ETCs	• Augments the I_*H*_ response following hyperpolarization (mice)	–
SACs	–	• May act as an autoreceptor
M/TCs	• Indirectly inhibits M/TCs by increasing feedforward inhibition from GCs (rats)	• Enhances GABA/Cl^–^ current (rats) • Inhibits glutamate release from apical dendrites (rats) • Directly hyperpolarizes mitral cells (fish)
		• Decreases mitral cell spontaneous spiking (frogs)
GCs	• Reduces GABA_*A*_/Cl^–^ currents (rats)	• Indirectly reduces excitation of these neurons by inhibiting the M/TCs (frogs)
Astrocytes	• Increases cytoplasmic Ca^2+^ (mice)	• Increases cytoplasmic Ca^2+^ (mice)

*No reported effects from D_3_-D_5_Rs were found. The symbol “–” indicates that there is no known effect of the D_1_ or D_2_ receptor on the particular cell type. All references for listed effects are described in section “Dopamine’s Neuromodulation of the Olfactory Bulb.” ETCs, external tufted cells; GCs, granule cells; M/TCs, mitral/tufted cells; OSNs, olfactory sensory neurons; PGCs, periglomerular cells; SACs, short-axon cells.*

Each of the couple of thousand of OB glomeruli receives a unique odor signal – that pass from OSNs to M/TCs, while also being modulated by the JGCs – which allows the OB to code for specific odors (Kratskin and Belluzzi, 2003). Once activated, dopamine is released from JGCs and binds D_2_Rs (as well as its GABA binding the metabotropic GABA_B_ receptor) on OSN terminals (Nickell et al., 1994; Hsia et al., 1999; Aroniadou-Anderjaska et al., 2000; Berkowicz and Trombley, 2000; Ennis et al., 2001; Liu et al., 2013; Vaaga et al., 2017) and the apical dendrites of M/TCs (Davila et al., 2003), causing a decrease in the presynaptic release of glutamate. Thus, D_2_R activation leads to an attenuation of postsynaptic excitation. This “inhibition of excitation” is likely caused by the D_2_R activating the G_βγ_ protein, which inhibits the N-type Ca^2+^ channels (Meir et al., 2000; Davila et al., 2003; Bettler et al., 2004). The N-type Ca^2+^ channels are involved in the vesicular release cascade (Miller, 1987; Weber et al., 2010), which may gate glutamate release from the OSN and M/TCs (Isaacson and Strowbridge, 1998). Additionally, D_2_Rs increase the GABA/Cl^–^ currents in M/TCs (Brünig et al., 1999), providing further inhibition to odor signal transmission. These inhibitory mechanisms may also occur via interglomerular dopamine and GABA release.

The activity of OB D_2_R was hypothesized to be a part of the high-pass filter function of dopamine neurons (Ennis et al., 2001; Korshunov et al., 2017, 2020). That is, these inhibitory/GABAergic dopamine neurons inhibit (through the metabotropic D_2_/GABA_B_R or ionotropic GABA_A_R) or “gate out” the weak, background odor stimulus (e.g., odor of rat bedding), but not the strong, prominent odor stimulus (e.g., predator’s urine), which is passed along to the M/TCs and to further brain regions. Our findings that a subset of OB dopamine neurons more actively spike in response to weak but not strong current stimuli (Korshunov et al., 2020) suggest that these neurons are more likely to inhibit the weak odors but be unresponsive to strong odors. Additionally, D_2_R activation hyperpolarized the mitral cells in the fish OB and made them less responsive to weak stimuli, while also making them more responsive to strong stimuli (Bundschuh et al., 2012). In general, dopamine application produces reduced responses of M/TCs in several species (Nowycky et al., 1983; Duchamp-Viret et al., 1997; Berkowicz and Trombley, 2000; Davison et al., 2004), and naris occlusion (Guthrie et al., 1990, 1991; Wilson and Sullivan, 1995) or the absence of D_2_Rs (Tillerson et al., 2006) produce the complete opposite effects. These mechanisms would conceivably increase odor discrimination and odor detection (Doty and Risser, 1989).

There is also some controversy about the effectiveness of D_2_Rs in presynaptic inhibition (McGann, 2013). While there is a clear effect of exogenous dopamine D_2_R agonists, the role of endogenously released dopamine on presynaptic inhibition has been difficult to establish (McGann, 2013). Maher and Westbrook (2008) perhaps came closest to studying endogenous dopamine activity in mammalian OB by applying cocaine (dopamine reuptake blocker), which reduced postsynaptic currents from OSNs. This effect that was reversed with the application of the D_2_R antagonist sulpiride (Maher and Westbrook, 2008). However, they were unable to observe the same decrease in OSN postsynaptic excitation when stimulating dopamine neurons directly (Maher and Westbrook, 2008). The effects of sulpiride by itself (without exogenous dopamine or D_2_R agonists) did not reverse the decrease of postsynaptic OSN excitation, suggesting that tonic dopamine release may be low (McGann, 2013). Conversely, GABA may be more relevant in this presynaptic inhibitory role, because activation of the GABA_B_R (which are also present on the OSN terminals) was able to suppress the synaptic mechanisms of OSNs (Wachowiak et al., 2005). Thus, the definitive role of dopamine/D_2_Rs in presynaptic inhibition remains to be elucidated.

While not as much is known about the effects of D_1_Rs, its activity has been implicated in increasing odor detection (Doty et al., 1998), which contrasts with the effects of D_2_R (Doty and Risser, 1989). Interglomerular dopamine neurons also act upon distant ETCs (Liu et al., 2013). Dopamine and GABA release first act to inhibit the ETCs via the GABA_A_R, which activates the hyperpolarization-activated I_H_ (Liu et al., 2013). Dopamine, which acts on a slower time course than GABA (Borisovska et al., 2013), further primes the I_H_ current by activating the D_1_R, causing rebound spiking in the ETCs (Liu et al., 2013). The D_1_R also indirectly inhibits M/TCs. Activating D_1_R on the inhibitory GCs inhibits their GABA_A_ current, causing an increase in the feedforward inhibition to the M/TCs (Brünig et al., 1999). Lastly, unlike the retina, it is not known whether D_1_ or D_2_Rs affect the OB gap junctions, of which Cx36 specifically couples the dendrites of mitral cells (Christie and Westbrook, 2006). Given that D_1_R activation uncouples Cx36 gap junctions between the AII ACs in the retina (Kothmann et al., 2009), a similar or conserved mechanism may exist in the OB, but further studies are needed.

## Parkinson’s Disease

One of the most common neurodegenerative diseases, PD, is characterized by a progressive loss of dopaminergic neurons in the substantia nigra pars compacta (SNc). Based on a meta-analysis that analyzed studies from 1985 to 2010 from around the world, the prevalence of PD increases with age and varies with location, with a higher prevalence seen in the 70–79-year age-group in North America, Europe, and Australia compared to Asia (Pringsheim et al., 2014). The most common symptoms of PD are motor disturbances (e.g., bradykinesia, tremors) (Carlsson, 1972), but non-motor disturbances are also present (Modugno et al., 2013), including disrupted sleep (Lima et al., 2007; Chaudhuri and Schapira, 2009; Lima, 2013; Rodrigues et al., 2014), prolactin cycling (Winkler et al., 2002), and circadian activity (Anderson and Maes, 2014; as reviewed by Korshunov et al., 2017), and even visual (Urwyler et al., 2014) and olfactory (Doty, 2012) disruptions occur. These symptoms may provide a clinical relevance to the neuromodulatory effects of dopamine in the retina and olfactory bulb.

### Visual Symptoms and Potential Impact of Retinal Dopamine Neurons

Like in other mammalian species, dopamine neurons are present in the INL of human retinas (Frederick et al., 1982). In human patients, optical coherence tomography examinations determined that the INL was significantly thinner in patients with PD than in control subjects (Hajee et al., 2009). Dopaminergic innervation is reduced in patients with PD (Nguyen-Legros, 1988) and in retinas of postmortem patients with PD (Harnois and Di Paolo, 1990). Interestingly, the retinas of patients with PD who received L-DOPA treatment shortly before passing had a similar number of dopamine neurons compared to non-Parkinsonian retinas (Harnois and Di Paolo, 1990). These findings show that PD can have physiological impacts on the human retina and retinal dopamine neurons, but what are some potential symptoms that could arise from this?

Along with some commonly reported symptoms (e.g., hallucinations, double vision, and text moving while attempting to read) (Urwyler et al., 2014), visual symptoms in patients with PD also include deficits in color discrimination and contrast sensitivity, which tend to worsen with the progression of the disease (Price et al., 1992; Tagliati et al., 1996; Diederich et al., 2002). The electroretinogram (ERG) is a common electrophysiological technique that can identify these symptoms by examining the depolarizing response of the ON-BCs to light stimuli (commonly referred to as the b-wave). Clinical studies using this technique on patients (Bodis-Wollner et al., 1987; Price et al., 1992; Hutton et al., 1993) and animals (Onofrj and Bodis-Wollner, 1982; Bodis-Wollner, 1997) confirm that contrast sensitivity (the ability to distinguish one object from another) is affected significantly in subjects with PD. Amazingly, contrast sensitivity in people was also improved after L-DOPA treatment (Harnois and Di Paolo, 1990; Hutton et al., 1993; Peppe et al., 1998). These results may indicate that dopaminergic neurons that are impacted in Parkinsonian retinas are no longer able to decrease the receptive fields of various neurons by uncoupling them, potentially leading to decreased contrast sensitivity Additionally, these visual symptoms appeared to not be a co-morbidity of general motor symptoms, because patients who had non-Parkinsonian physical lesions to their basal ganglia did not experience these visual symptoms (Peppe et al., 1998).

While it is clear that there is some symptomatic impact of PD on the mammalian retina, it is important to also note that visual symptoms of PD can arise beyond the retina. For example, orientation-specific stimuli, which are processed by the visual cortex, are also impacted in PD (reviewed in Weil et al., 2016).

### Olfactory Symptoms and Potential Impact of Olfactory Bulb Dopamine Neurons

Olfactory deficits (e.g., hyposmia and anosmia) are notable prodromal stages of PD that precede motor symptoms by years (Doty, 2012). In fact, non-motor smell tests are being encouraged to potentially diagnose PD before the emergence of motor symptoms (Berendse et al., 2001; Ponsen et al., 2004; Bohnen et al., 2008). These olfactory symptoms may result from impacted OB dopamine neurons. Paradoxically, dopamine neuron numbers in people with PD (Huisman et al., 2004; Mundiñano et al., 2011) and in Parkinsonian rodent models (Lelan et al., 2011) are dramatically higher. This increase may be part of a compensatory mechanism from the SVZ of rodents, where neurogenesis of OB dopamine neurons (related to olfactory recovery) occurred following traumatic brain injury (Marin et al., 2017). The reason for this increase in people with PD is not as clear. While it is originally thought that human OBs do not receive SVZ-derived neuroblasts, there is also evidence to the contrary (Bédard and Parent, 2004). Given that we and others have shown that OB dopamine acts as a presynaptic inhibitor of glutamate release from the OSNs (Nickell et al., 1994; Hsia et al., 1999; Berkowicz and Trombley, 2000; Ennis et al., 2001; Liu et al., 2013; Vaaga et al., 2017), and that increasing D_2_R activation can lead to a decrease in odor detection (Doty and Risser, 1989; Escanilla et al., 2009), it is likely that an increase in dopamine and D_2_R activity can lead to increased hyposmia and anosmia during the prodromal stages of PD (Huisman et al., 2004).

This research is ongoing, and recent studies are providing further insight (and potential inconsistencies) on the impacts of PD on the OB. Transgenic mice with ablated vesicular monoamine transported 2 (VMAT2, a protein that allows dopamine to be packaged into vesicles)-expressing neurons displayed higher olfactory deficits (even though dopamine was reduced) and lower neurogenesis (Ma et al., 2019). Studies that ablate OB dopamine neurons with 6 hydroxydopamine (6-OHDA, a common neurotoxin of dopaminergic neurons) show a similar decrease in olfaction (Höglinger et al., 2004; Ilkiw et al., 2019). Additionally, injection of 6-OHDA into the SNc dopaminergic neurons also decreased olfaction (Höglinger et al., 2015). This study is notable, because the authors report the first evidence of centrifugal dopaminergic innervation, stemming from the midbrain to the OB (Höglinger et al., 2015), which was previously reported not to exist. The authors mention that this decreased innervation is the primary cause of the symptoms. Thus, the mechanisms for olfactory symptoms in PD are complex, but likely involve some interplay between the OB and SNc dopamine neurons.

However, like the retina, olfactory impairments can stem beyond the OB (and the SNc). One such study found that there is an increase in inflammatory activity of microglia in the PC (Sancandi et al., 2018). This indicates that the impact of PD on vision and olfaction are not just localized to the retina and OB. It is for the benefit of patients and for those at risk of developing PD to better understand these impacted mechanisms, to hopefully bring about a clinical foundation for better treatment.

## Conclusion

Two of the arguably most crucial senses of the vertebrate world are vision, which is processed by the retina, and olfaction, which is processed by the OB. Whereas one area receives electromagnetic stimuli and the other chemical stimuli, once these stimuli are transduced into electrical pulses, the retina and OB become remarkably similar. For example, both areas process sensory signals by a sequential order of neurons embedded in distinct laminae, both areas utilize lateral inhibition, and both areas are affected by the well-known neuromodulator dopamine. Thus, dopamine is not only important for the study of addiction and movement disorders, but it is also a crucial component of visual and olfactory processing.

In this paper, we have reviewed the decades-old to brand new literature of dopamine’s influence on the retina and OB, focusing on similarities of these structures that are otherwise functionally distinct. The morphology of dopamine neurons in both structures is heterogenous, and their identities are not completely agreed upon. The retinal dopamine neurons help maintain the autonomous circadian rhythmicity in the retina, while the value of the OB dopamine neurons to the bulb’s circadian rhythm is yet to be determined. Both sets of neurons increase their dopamine synthesis and activity in response to light or olfactory stimuli, and both sets of neurons have various biophysical responses to different intensities of these stimuli. The neuromodulatory mechanisms of both retinal and OB dopamine neurons are complex, and they are crucial for mediating the transition from nighttime to daytime processing in the retina and for maintaining the odor threshold and discrimination in the OB. Several symptoms of PD, including decreased contrast sensitivity and increased hyposmia and anosmia, could very well be due to the dysregulation of these neuromodulatory effects of dopamine.

Overall, while the role of dopamine continues to be illuminated and sniffed out in the retina and OB, respectively, there are many questions that remain to be answered. These questions also relate to the many potential differences between these two structures. What activates dopamine synthesis in neurons deep to the GL in the OB? While there is some evidence that dopamine neurons in the EPL can already receive excitatory synapses from the OSNs (Pignatelli et al., 2009), do other excitatory synapses influence the expression of TH in OB dopamine neurons? Furthermore, do these neurons exhibit other markers (besides a high intracellular Cl^–^ concentration) that would make them immature (Pignatelli et al., 2009)? With new evidence that anaxonic, Small OB dopamine neurons are born postnatally (Galliano et al., 2018), are all deep OB dopamine neurons anaxonic? Whereas small, anaxonic subtypes of dopamine neurons in the OB continue to be generated throughout life, preliminary evidence (in fish) that some retinal dopamine neurons can regenerate after chemical ablation (Hitchcock and Vanderyt, 1994) also requires further investigation. What is the exact function of the Type-2 catecholamine/epinephrine neuron in the retina (Zhang et al., 2004), and are these neurons lost with age or is their expression of TH decreased (Wang et al., 1990)? Does dopamine in the OB influence the autonomous inner clock, like it does in the retina (Ruan et al., 2006, 2008)? What synaptic inputs regulate the activity of the retinal dopamine neurons that do not respond to light (Zhang et al., 2007)? How may the proposed dopaminergic input from the midbrain influence odor discrimination and other olfactory functions (Höglinger et al., 2015)? Lastly, how does dopamine affect glial cells, and how may this influence vision and olfaction? There is already evidence of OB astrocytes increasing their Ca^2+^ activity in response to the activation of D_1_ and D_2_Rs (Fischer et al., 2020), which is a fascinating note on which to end, because it implies that dopamine in the retina, OB, basal ganglia, and other brain areas would not only affect the neurons, but perhaps every type of brain cell.

## Author Contributions

KK and LB performed the literature search and review. KK wrote the first draft of the manuscript. LB and PT edited and contributed to all subsequent drafts. All authors agreed upon the final version of this manuscript.

## Conflict of Interest

The authors declare that the research was conducted in the absence of any commercial or financial relationships that could be construed as a potential conflict of interest.

## Abbreviations

6-OHDA: 6-hydroxydopamine
AC: amacrine cell
cAMP: cyclic adenosine monophosphate
D_1_R: dopamine receptor 1
D_2_R: dopamine receptor 2
D_3_R: dopamine receptor 3
D_4_R: dopamine receptor 4
D_5_R: dopamine receptor 5
DAT: dopamine transporter
EPL: external plexiform layer
ERG: electroretinogram
ETC: external tufted cell
GABA: γ-aminobutyric acid
GAD-65: glutamic acid decarboxylase 65
GAD-67: glutamic acid decarboxylase 67
GC: granule cell
GCL_OB_: granule cell layer
GCL_R_: ganglion cell layer
GFP: green fluorescent protein
GL: glomerular layer
HC: horizontal cell
HIOMT: hydroxyindole-O-methyltransferase
I_Ca_: Ca^2+^ current
I_H_: h-current
I_K_: K^+^ current
I_Na_: Na^+^ current
INL: inner nuclear layer
IPL: inner plexiform layer
ipRGC: intrinsically photosensitive retinal ganglion cell
JGC: juxtaglomerular cell
M/TC: mitral and/or tufted cell
MCL: mitral cell layer
NAT: serotonin N-acetyltransferase
Na_V_: voltage-gated Na^+^ channel
OB: olfactory bulb
ONL_OB_: olfactory nerve layer
ONL_R_: outer nuclear layer
OPL: outer plexiform layer
PC: piriform cortex
PD: Parkinson’s disease
PGC: periglomerular cell
PKA: protein kinase A
RGC: retinal ganglion cell
RMS: rostral migratory stream
SAC: short axon cell
SCN: suprachiasmatic nucleus
SNc: substantia nigra pars compacta
TH: tyrosine hydroxylase
VIP: vasoactive intestinal polypeptide
VMAT2: vesicular monoamine transporter 2.
